# A rare loss-of-function genetic mutation suggest a role of dermcidin deficiency in hidradenitis suppurativa pathogenesis

**DOI:** 10.3389/fimmu.2022.1060547

**Published:** 2022-12-05

**Authors:** Paola Maura Tricarico, Rossella Gratton, Carlos André dos Santos-Silva, Ronald Rodrigues de Moura, Blendi Ura, Eduardo Sommella, Pietro Campiglia, Cecilia Del Vecchio, Chiara Moltrasio, Irene Berti, Adamo Pio D’Adamo, Ahmed M. A. Elsherbini, Lena Staudenmaier, Karin Chersi, Michele Boniotto, Bernhard Krismer, Birgit Schittek, Sergio Crovella

**Affiliations:** ^1^ Department of Advanced Diagnostics, Institute for Maternal and Child Health - IRCCS Burlo Garofolo, Trieste, Italy; ^2^ Maternal-Neonatal Department, Institute for Maternal and Child Health - IRCCS Burlo Garofolo, Trieste, Italy; ^3^ Department of Pharmacy, University of Salerno, Salerno, Italy; ^4^ Dermatology Unit, Fondazione IRCCS Ca’ Granda Ospedale Maggiore Policlinico, Milan, Italy; ^5^ Department of Medical Surgical and Health Sciences, University of Trieste, Trieste, Italy; ^6^ Pediatric Department, Institute of Maternal and Child Health - IRCCS Burlo Garofolo, Trieste, Italy; ^7^ Department of Infection Biology, Interfaculty Institute of Microbiology and Infection Medicine, University of Tübingen, Tübingen, Germany; ^8^ Department of Dermatology, Division of Dermato-oncology, University of Tübingen, Tübingen, Germany; ^9^ Dermatological Clinic, ASUGI - Azienda Sanitaria Universitaria Giuliano Isontina, Trieste, Italy; ^10^ INSERM, IMRB, Translational Neuropsychiatry, University Paris Est Créteil, Créteil, France; ^11^ Department of Biological and Environmental Sciences, Biological Sciences Program, College of Arts and Sciences, University of Qatar, Doha, Qatar

**Keywords:** hidradenitis suppurativa, dermcidin, genetics, antimicrobial peptides, bacteria

## Abstract

Hidradenitis suppurativa (HS) is a chronic inflammatory skin disease with a multifactorial aetiology that involves a strict interplay between genetic factors, immune dysregulation and lifestyle. Familial forms represent around 40% of total HS cases and show an autosomal dominant mode of inheritance of the disease. In this study, we conducted a whole-exome sequence analysis on an Italian family of 4 members encompassing a vertical transmission of HS. Focusing on rare damaging variants, we identified a rare insertion of one nucleotide (c.225dupA:p.A76Sfs*21) in the *DCD* gene encoding for the antimicrobial peptide dermcidin (DCD) that was shared by the proband, his affected father and his 11-years old daughter. Since several transcriptome studies have shown a significantly decreased expression of DCD in HS skin, we hypothesised that the identified frameshift insertion was a loss-of-function mutation that might be associated with HS susceptibility in this family. We thus confirmed by mass spectrometry that DCD levels were diminished in the affected members and showed that the antimicrobial activity of a synthetic DCD peptide resulting from the frameshift mutation was impaired. In order to define the consequences related to a decrease in DCD activity, skin microbiome analyses of different body sites were performed by comparing DCD mutant and wild type samples, and results highlighted significant differences between the groins of mutated and wild type groups. Starting from genetic analysis conducted on an HS family, our findings showed, confirming previous transcriptome results, the potential role of the antimicrobial DCD peptide as an actor playing a crucial part in the etio-pathogenesis of HS and in the maintenance of the skin’s physiological microbiome composition; so, we can hypothesise that DCD could be used as a novel target for personalised therapeutic approach.

## 1 Introduction

Hidradenitis Suppurativa (OMIM #142690; HS), also known as acne inversa, is a chronic-relapsing, debilitating autoinflammatory skin disease of the pilosebaceous unit, with a prevalence in Europe of 0.8%, ranging from 0.5 to 1.3% (1). It typically occurs during puberty or early adulthood (from the second or third decade of life) and is clinically characterized by painful, deep-seated, recurrent nodules often evolving into abscesses and sinus tracts - these latter containing “invasive proliferative gelatinous mass” composed of immune cells, cytokines, extracellular traps and matrix metalloproteinases - (2), with disfiguring hypertrophic scarring, mainly occurring in apocrine gland bearing skin (3).

HS is usually a sporadic disease, however, 40% of reported cases reveal a positive family history of the disorder with an autosomal dominant, although with incomplete penetrance, mode of inheritance (3). Despite the abundance of familial cases, three morbid genes have been identified so far and encompass *NCSTN*, *PSEN1* and *PSENEN* genes encoding for 3 out of the 4 components of the γ-secretase, a multi-protein complex that catalyses the intramembrane proteolysis of various substrates associated to diverse intracellular signalling routes primarily involved in the regulation of processes including cellular differentiation, proliferation, migration and cell fate (4, 5). Apart from the typical pool of mutations occurring in *NCSTN*, *PSEN1* and *PSENEN*, other susceptibility genes potentially linked to HS have been currently recognised (6, 7). Nevertheless, due to the fact that the identified genetic variations in HS familial cases are rare and mostly given by private mutations, they fail to explain the genetic basis underlying familial forms of HS, hence further studies aimed at identifying novel genetic variants and genes potentially associated with the disease are strictly required (8).

HS is a complex disorder with a multifactorial aetiology that involves a strict interplay between genetic, immunologic and environmental risk factors (e.g. obesity and tobacco smoking). The combination of these events collectively lead to a cutaneous microbial dysbiosis and to a deregulated immune activation around terminal hair follicles in intertriginous body areas, resulting in the plugging, rupture and scattering of the follicular content in the surrounding dermis (9, 10).

The occurrence of aberrant immune responses in HS is tightly associated to an exaggerated activation of cellular components of both the innate and adaptive immune system principally resulting in an augmented production of inflammatory cytokines and of cutaneous antimicrobial peptides (AMPs) (9, 11, 12).

Specifically, AMPs are considered as key effectors of cutaneous innate immune responses, and in the skin compartment the majorly expressed ones include defensins, cathelicidins, dermcidin, other small peptides, chemokines and psoriasin (13). In general, the antimicrobial activity of AMPs is aimed at counteracting bacterial propagation, function exerted either by disrupting the structural integrity of the membrane of pathogens or by interacting with diverse intracellular components following their internalisation in bacterial cells (14). AMPs are normally constitutively expressed in human epithelia mainly by keratinocytes, neutrophils, sebocytes or sweat glands; nevertheless, following an inflammatory stimulus their expression can be rapidly enhanced (15).

According to a recent hypothesis, a deregulation in AMPs production may be responsible for the altered growth of the skin’s microflora, condition that might trigger a dysbiosis-driven activation of the innate immune system that can further sustain the perpetuation of inflammatory responses in HS skin (12). In particular, recent studies on skin transcriptome showed a dysregulation of AMPs in HS patients, compared to healthy controls; a downregulation of a specific skin AMPs naturally found in human sweat, dermcidin (DCD) has been reported (11, 16).

Dermcidin is expressed and secreted by eccrine sweat glands and after proteolytic processing it showed to be active against both Gram-positive and Gram-negative bacteria. DCD actively participates in cutaneous innate immune defence, low levels of this AMP have been associated with conditions like atopic dermatitis or hidradenitis suppurativa (11, 16–18).

The active moiety of DCD is composed by 48 amino acids presenting a well-defined amphiphilic structure organised in a cationic N-terminal region (Ser1-Lys23) and an anionic C-terminal region (Asp24-Leu48) (19). The antimicrobial activity of DCD is thought to occur *via* initial interactions of the cationic N-terminal domain with the negatively charged bacterial phospholipids, subsequent clustering of DCD peptides on the bacterial surface ultimately resulting in the formation of membrane-embedded ion channels delineated by the N-terminal domains, while the anionic C-terminal region seems to be involved in the stabilisation of the complex (17).

The role of bacterial infections in HS has for long been debated and still needs to be fully defined. However, HS is currently not considered as a primary infectious disease but rather as a condition in which bacterial infections are thought to act as boosters and amplifiers further eliciting cutaneous inflammatory responses (20, 21). Though the mechanisms at the basis of these interactions are still not clear, further investigations aimed at unravelling the role of bacterial dysbiosis in the pathogenesis of HS are strictly required due to the registered high efficacy of antibiotic treatments in the clinical practice and to promising preliminary published results (22, 23).

In this study, whole exome sequencing analysis conducted on a family affected by HS allowed the identification of a rare frameshift insertion in *DCD* gene, encoding for the AMP dermcidin (DCD), in affected individuals. In order to initially determine the effect of the identified genetic alteration, *in silico* studies together with the quantification of DCD levels in sweat samples derived from patients and healthy controls were performed. Next, considering the well-established role of AMPs in HS skin biology, we also decided to assess microbiome analysis of patients and healthy controls and to evaluate the antimicrobial activity of the mutant peptide with respect to the wild type moiety.

## 2 Materials and methods

### 2.1 Patients

A 52-year-old male (Patient 1) came to our attention complaining of persistent inflamed nodules, some of which coming to abscesses, under both armpits, in the suprapubic and inguinal regions, in the left buttock and on the right ear lobe. A clinical diagnosis of HS was made with an IHS4 score of 9, corresponding to a moderate form of the disease. The patient reported an age of HS onset at about 23 years with the development of inflamed nodules in the inner thighs, inguinal and cervical regions. Moreover, the patient was overweight (29 body mass index), smoker, and presented comorbidities including psoriasis, celiac disease and hyperlipidaemia. Tetracycline (300 mg daily) and clindamycin ((600 mg daily) were administered during the diseases’ flares. He also reported a positive family history for HS: his 77-year-old father (Patient 2) was also affected by HS with the occurrence of HS lesions mainly located on inguinal and ano-genital regions, for which no topical or systemic treatment was administered. In his 11-year-old daughter, follicular lesions appeared once, both in the perineal and inguinal areas, but resolved spontaneously and without flares. All participating individuals signed a written informed consent previously approved by the Comitato Etico Unico Regionale (CEUR) of Friuli Venezia Giulia (FVG) (RC 16/18, Prot. N.0001094 (14/01/2019), CEUR-2018-Sper-127-BURLO).

### 2.2 Genomic DNA extraction and whole exome sequencing

Genomic DNA was extracted from saliva samples using the Oragene-DNA (Oragene^®^, Ottawa, Canada) kit following the manufacturer’s instructions. Agarose gel (2%) and Qubit instrument (Invitrogen^®^, Oregon USA) were used to evaluate DNA quantity and quality prior to sequencing.

Whole exome sequencing (WES) was outsourced and performed by Macrogen Europe (Amsterdam, Netherlands). The Exome Sequencing Analysis, with a declared 150× coverage, used the Illumina^®^ SureSelect Human V7 Kit Library preparation and sequencing reaction in an Illumina^®^ HiSeq 2500 System, generating pair-end reads of 150 base pairs.

### 2.3 WES data analysis

WES analysis has been performed using the InterOMICs Genome Pro software, as described in our previous study (24, 25). In synthesis, raw fastq.gz data were checked using fastQC (26), where parameters like average read length, read and base quality scores, and presence of adapters were assessed. Then, residual adapters, short reads (below 25 base pairs) and/or low-quality reads (Q < 20) were removed using TrimGalore (27).

Unmapped reads were aligned with NCBI GRChg38 reference genome using BWA algorithm (Li and Durbin); marking and removal of duplicated reads as well as base quality score recalibration was carried out using Picard Tools v.2.7.0 and GATK v.4.1.2.0, respectively (28, 29).

Variant calling was made using Strelka2 (30) and variant annotation using Annovar software (31). Candidate variants were selected according to the following filters: been heterozygous for both affected individuals and wildtype for non-affected individual; exonic; non-synonymous, stop gain/loss, splicing site variants or frameshift indels; minor allele frequency lower than 1% according to GnomAD or 1000 Genomes Project; damaging, possible/probably damaging or conflicting evidence about pathogenicity according to CLINVAR.

Finally, WES results were validated by Sanger Sequencing.

### 2.4 *In silico* analysis

#### 2.4.1 Comparative modelling

For three-dimensional (3D) modelling purposes, an alignment of each sequence was made against the Protein Data Bank (PDB) using the BLASTp tool to find the best template protein for constructing comparative models, where the defensins with the highest sequence identity and complete coverage of the conserved domain were selected. Using Modeller 10.1 (32) one hundred models were constructed for each dermcidin sequence. The best model was chosen according to the discrete optimised protein energy (DOPE) score that assesses the model’s energy and indicates the most probable structure. Finally, the models were validated by analysing the quality of the folding and stereochemistry of the models. Structural features of dermcidin based on conserved sites were accessed by comparing 3D models using PyMOL software.

#### 2.4.2 Molecular dynamics

Simulations were conducted with the GROMACS 2019.4 package. The dermcidin modelled in the previous step were placed in the center of a cubic box and solvated using the Simple Point Charge water model (33). The system was solvated in NaCl solution at a physiological concentration (0.15 M), replacing solvent molecules with Na^+^ and Cl^-^ ions, followed by energy minimization. The temperature was maintained at 300 K in the NvT ensemble with solute atoms constrained in the initial position. The LINCS method (34) was used to constrain bonds involving hydrogen atoms. Integration was carried out by the leapfrog algorithm using a 2-fs integration time step. The systems were initially optimised for energy using 50,000 steps of the steepest descent algorithm. All atomistic simulations were performed for 100 ns using the GROMOS 53A6 (35) force fields and periodic boundary conditions in the x, y, and z directions. Finally, the molecular dynamics was performed unrestrained both at a constant pressure and temperature respectively of 1 atm and 300 K.

#### 2.4.3 Principal component analysis

In order to establish the main conformational changes of the systems during the simulation in molecular dynamics, principal component analysis (PCA) was performed using the trajectories of performed simulations with the GROMACs package (anaeig). The technique was assessed for α-carbon atoms in a 100 ns interval during the dynamics simulation. The Cartesian coordinates of such atoms were used to generate the covariance matrix and the first two components were used to develop the graphic result.

### 2.5 Determination of DCD levels in sweat

Human sweat was collected from thirteen healthy volunteers, from the proband and his father. Participants were asked neither to wash nor to apply topical treatments in the 6 hours preceding sampling. Before sweat sampling, the tested skin region (2 × 2 inches) was washed with 70% ethanol, rinsed with ultrapure water and dried using filter paper. Sweating was induced by iontophoresis using pilocarpine gel-padded electrodes with an electric current of 5 mA for 5 minutes. After stimulation, sweat was collected on a filter paper from the forearm for 30 minutes. The filter paper was then placed in a laboratory dish of known weight so the quantity of the collected sweat can be calculated (36). The sweat was then separated from the filter paper *via* elution for 1 hour at room temperature and immediately centrifuged for 1 minute at 13,000 rpm to remove particles and debris and then frozen at -80°C. The protein sweat content was determined using the Bradford assay. Shortly, proteins were resuspended in 20 μL of 0.5% trifluoroacetic acid (TFA) in 5% acetonitrile (ACN) and processed with Pierce C18 spin Columns.

DCD wild type (DCD_WT) and DCD mutated (DCD_MT) peptides at >95% purity have been purchased from NovoPro Bioscience Inc. (Shanghai, China) and resuspended in HCl 10 mM, vacuum-dried and then resuspended in H_2_O. The quality and purity of peptides was verified by ESI-MS (API 150 EX Applied Biosystems). The concentration of each stock solution was evaluated by spectrophotometric determination of tryptophan by measuring the differential absorbance at 215 nm and 225 nm (37), and by spectrophotometric determination of peptide bonds (ϵ214 calculated as reported by Kuipers and Gruppen) (38).

For proteomic analysis the trypsin digestion was performed as follows: samples were solubilized in 50 mM ammonium bicarbonate, vortexed gently, proteins were reduced with 10 mM dithiothreitol (DTT) for 30 minutes and then alkylated with 20 mM iodoacetamide (IAA) for 1 hour in the dark. Enzyme-substrate ratio was 1:100 and the sample was incubated at 37°C overnight in a thermomixer comfort (Eppendorf). Next, following centrifugation, the supernatant was collected and employed for mass spectrometry (MS) analysis. A nanoflow ultra-high performance liquid chromatography (UHPLC) instrument (Ultimate 3000, Thermo Fisher Scientific, Bremen, Germany) was coupled on-line to an Orbitrap Lumos tribrid mass spectrometer (Thermo Fisher Scientific) with a nanoelectrospray ion source (Thermo Fisher Scientific). 1 μL of digest was initially trapped on a PepMap trap column for 1.5 minutes at a flow rate of 30 L/min (Thermo Fisher) and then peptides were loaded and separated onto a C18-reversed phase column (25 cm x 75 μm I.D, 2.6 m, BioZen Phenomenex, Bologna, Italy). Mobile phases were 0.1% HCOOH in water (A), 0.108% HCOOH in ACN/Water (80/20, *v*/*v*) (B), a linear 60 minute gradient was performed.

Each sample was analysed by LC-MS/MS in triplicate and results are expressed as relative intensity ± SD. Specifically, MS data were acquired using a data-dependent method dynamically choosing the most abundant precursor ions from the survey scan (350–1500 m/z) using HCD fragmentation. Survey scans were acquired at a resolution of 120,000 at m/z 200. Unassigned precursor ion charge states as well as singly charged species were excluded. Isolation window was set to 3 Da normalised collision energies (NCE) of 27 was applied. Maximum ion injection times for MS (Orbitrap) and the MS/MS (Ion Trap) scans were 50 ms and 100 ms respectively, and ACG values were set to auto. Dynamic exclusion: 30 seconds. For data processing, raw MS data were analysed using Proteome Discoverer software version 2.5 using the Sequest search engine. Maximum allowed mass deviation was set to 10 ppm. Enzyme specificity was set to trypsin; two missed cleavages were allowed. Carbamidomethylcysteine was set as a fixed modification, and methionine oxidation as variable modifications. The spectra were searched against the *Homo sapiens* (Human) sequence database from SWISSPROT database 11/2022, taxonomy id 9606) combined with 248 common contaminants and concatenated with the reversed versions of all sequences.

### 2.6 *In vitro* antimicrobial assays

#### 2.6.1 Bacterial strains and culture conditions

*Staphylococcus aureus (S. aureus* USA300LAC wild type*), Staphylococcus epidermidis* (*S. epidermidis* 1457) and *Staphylococcus lugdunensis* (*S. lugdunensis* IVK28) were preserved in 40% glycerol and 60% tryptic soy broth (TSB, Sigma-Aldrich) and stored at -80°C. Two days before the determination of the antimicrobial activity of DCD peptides on planktonic bacteria, on biofilm formation and on pre-formed biofilm, bacterial strains were seeded on tryptic soy agar (TSA, Sigma-Aldrich) plates and grown at 37°C overnight. The day prior to antimicrobial testing, for each strain one colony was collected from TSA plates using a sterile 10 μL microloop, resuspended in TSB and incubated at 37°C overnight with orbital shaking.

#### 2.6.2 *In vitro* antimicrobial activity of DCD peptides

An initial standardised inoculum presenting a final test concentration of 1 x 10^8^ colony forming units (CFU)/mL was prepared for each tested bacterial strain. Next, every bacteria specimen was incubated with DCD_WT or DCD_MT peptides at the final concentration of 2 μM (20 μg/mL). After 3 hour incubation, 10-fold serial dilutions of bacterial suspensions were seeded on TSA plates and the number of CFUs were counted following incubation at 37°C overnight.

#### 2.6.3 *In vitro* activity of DCD peptides against biofilm formation

The inhibitory effect of DCD peptide on biofilm formation was assessed against *S. aureus*. In brief, 10 μl of a standardised inoculum presenting a final concentration of 1-5 x10^7^ CFU/mL was added to wells of a 96-well flat-bottom non-treated tissue-culture microtiter plate (Sarstedt, Germany) containing 100 μL of Brain Heart Infusion (BHI) broth (Sigma-Aldrich) enriched with 2% sucrose containing DCD_WT or DCD_MT peptide at a 2 μM (20 μg/mL) final concentration. Following incubation at 37°C for 24 hours, non-adherent bacteria were removed by washing twice each well with 100 μL of sterile PBS (pH 7.2; Sigma-Aldrich S.r.l.). In order to fix slime and adherent bacteria, cells were incubated with methanol (Sigma-Aldrich) for 15 minutes at room temperature. Next, cells were stained for 5 minutes at room temperature with 100 μL of 0,1% crystal violet solution (Sigma-Aldrich). Wells were then rinsed with distilled water and the solubilisation of crystal violet solution was obtained by adding 200 μL of 95% ethanol at room temperature for 30 minutes under shaking conditions. The absorbance reads were acquired at 560 nm using the GloMax^®^ Discover Microplate Reader (Promega). The low cut-off was represented by approximately 3 standard deviations above the absorbance mean of control wells (containing medium alone without bacteria).

### 2.7 Microbiome analysis

Microbiome samples were obtained by swabbing the anterior nares (N), axillae (A), groin (G) of the proband, his affected father, his daughter and five healthy volunteers with COPAN eSwabs 480CE (COPAN ITALIA, Italy). Concurrently, a microbiome sample from an active lesion (L) in the groin area was collected from the proband. Nares were swabbed by rotating the eSwab 4-5 times over the nasal mucosa, while the other target body sites were sampled by swabbing about 20 cm² of the dry skin with pre-wetted COPAN eSwabs. Swabs were stored in the provided AMIES transport medium, and all samples were processed within 6 hours after swabbing. Nevertheless, since the samples derived from HS patients had to be shipped from Italy to Germany for microbiome analysis, to the AMIES transport medium 1 mL glycerol was added. After freezing at -80°C, the samples were shipped in dry ice and processed immediately after receipt. Control experiments showed that freezing has no or only minor effects on the results of microbiome analysis. Following centrifugation of the samples, DNA was extracted from all pellets with the HostZERO™ Microbial DNA Kit (ZymoResearch Europe GmbH, Germany) according to the manufacturer’s specifications. DNA was eluted in ultrapure water and stored at -20°C. To achieve a better resolution at the species level, the V1-V3 region was amplified using primer pair 518F (forward primer 5’-CAATTACCGCGGCTGCTGG-3’) and 27R (reverse primer 5’-CCGAGTTTGATCMTGGCTCAG-3’) (10.1186/s40168-020-00841-w).

Library Preparation and Sequencing was performed by the Institute for Medical Microbiology (part of the NGS Competence Center NCCT (Tübingen, Germany). For library preparation the first step PCR was performed in 25 µL reactions including KAPA HiFi HotStart ReadyMix (Roche), 518F and 27R (16S V1-V3 region) and maximum volume of template DNA (PCR program: 95°C for 3’ followed by 28 cycles at 98°C for 20’’, 55°C for 15’’, 72°C for 15’’), and then 72°C for 5’). First, PCR products were purified using 28 µL AMPure XP beads and eluted in 50 µL 10 mM Tris-HCl. Indexing was performed in the second step PCR including KAPA HiFi HotStart ReadyMix (Roche, Switzerland), index primer mix (Illumina Nextera XT Index Kit v2), purified first PCR product as template (PCR program: 95°C for 3’, followed by 8 cycles at 95°C for 30’, 55°C for 30’’, 72°C for 30’’), and then 72°C for 5’). After another bead purification (20 µL AMPure XP beads, eluted in 30 µL 10 mM Tris-HCl) the libraries were checked for correct fragment length on an agarose gel, quantified with a Qubit dsDNA BR Assay Kit (Thermo Fisher Scientific, Germany) and pooled equimolarly. Sequencing was performed on an Illumina MiSeq device with v3 sequencing kit in run mode 101, 8, 8, 401 with 30% PhiX spike-in and a loading concentration of 13.5 pM.

#### 2.7.1 Sequence processing

Demultiplexed reads were checked for the primers’ presence using Cutadapt (v1.18) (39). Then, raw reads quality filtering/trimming, error rate learning, sample inference, pairs concatenations, ASVs calling and chimera removal were done using functions filterAndTrim(),learnErrors(), dada(), mergePairs(), makeSequenceTable(), removeBimeraDenovo() respectively implemented in DADA2(v1.22.0) (40) pipeline in R (v.4.1.3) environment (41).

Exported ASVs table was then imported in QIIME2 (v.2021.11) (42) environment as a biom table. Taxonomic assignment was performed employing Naive-Bayes classier trained as shown in (43).

To calculate core diversity metrics, alpha-rarefaction curves were concluded with a QIIME2 plugin. Then, using 1800 reads as a sequence depth for random sub-sampling, matrices were computed using Phyloseq (v.1.38) (44) and Microbial (v.0.0.20) (45) packages in R. Of note, alpha diversity metric was expressed (Inverse Simpson, Shannon and Chao1) and compared using Wilcoxon signed-rank test, while beta diversity metric was demonstrated using weighted UniFrac distance and Principal Coordinate Analysis (PCoA).

For differential abundance analysis, using Deseq2 Package (46), anatomical sites were compared separately (e.g.: Nasal DCD_WT vs. DCD_MT). Then, after adjustment of multiple testing using the default Benjamini-Hochberg (BH) adjustment, statistically significant taxa (P-value <0.05) were exported including log2fold change values. Finally, the taxonomic identity of differentially abundant taxa was confirmed by blasting its representative sequence against the NCBI database.

### 2.8 Statistical analysis

Data analysis was performed by ordinary one-way analysis of variance (ANOVA) followed by Dunn’s multiple comparisons test (*P<0.05; **P<0.01; ***P<0.001; ****P<0.0001), comparing non treated (control) cells with the other experimental conditions. Results represent at least three independent experiments with at least three technical replicates ± SD. Statistical analysis has been conducted with Prism 6.0 software (GraphPad Software, La Jolla, California, USA).

## 3 Results

### 3.1 WES results

**Figure 1:**
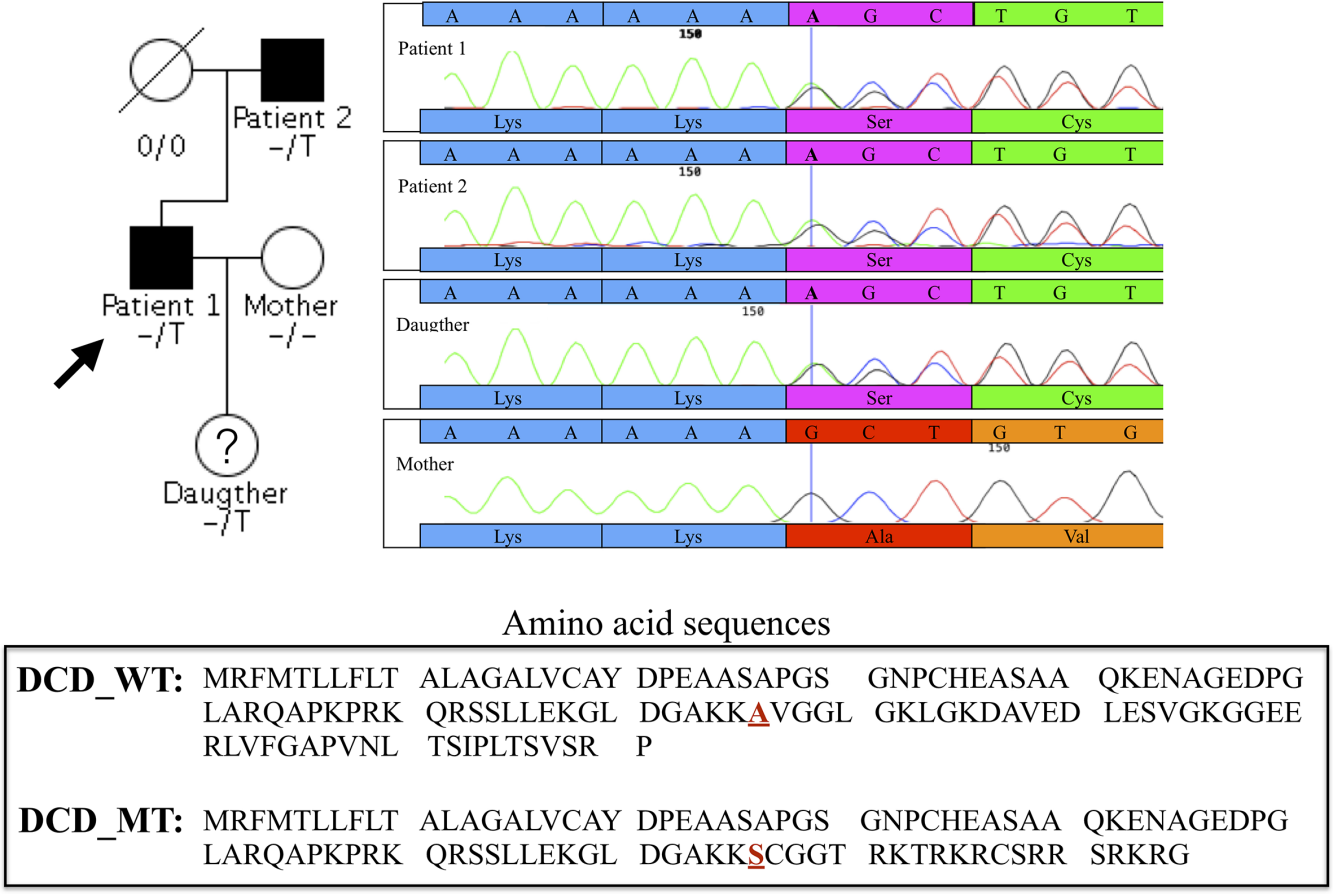
Family pedigree of the proband and chromatograms of the heterozygous single-nucleotide variation in exon 4 of DCD gene identified in the proband (Patient 1), Patient 2 and in the daughter, absent in the clinically healthy mother. The vertical bar in the electropherogram shows the site of rs538180888 insertion in exon 4, present in the two patients and the daughter but not in the mother.

Given the vertical transmission of HS in this small Italian family, we hypothesised a Mendelian autosomal dominant transmission of the disease. We found 12 damaging coding variants shared by the proband (Patient 1) and his father but not present in the proband’s wife (**Supplementary Table 1**). Considering the function of the proteins encoded by the 12 genes carrying these variants, we decided to focus our attention on a 1- base pair (bp) insertion in the exon 4 of the *DCD* gene. This variant is present in heterozygous state in the proband, his affected father and his 11 year-old daughter (**Figure 1**).

### 3.2 DCD levels in sweat

**Figure 2:**
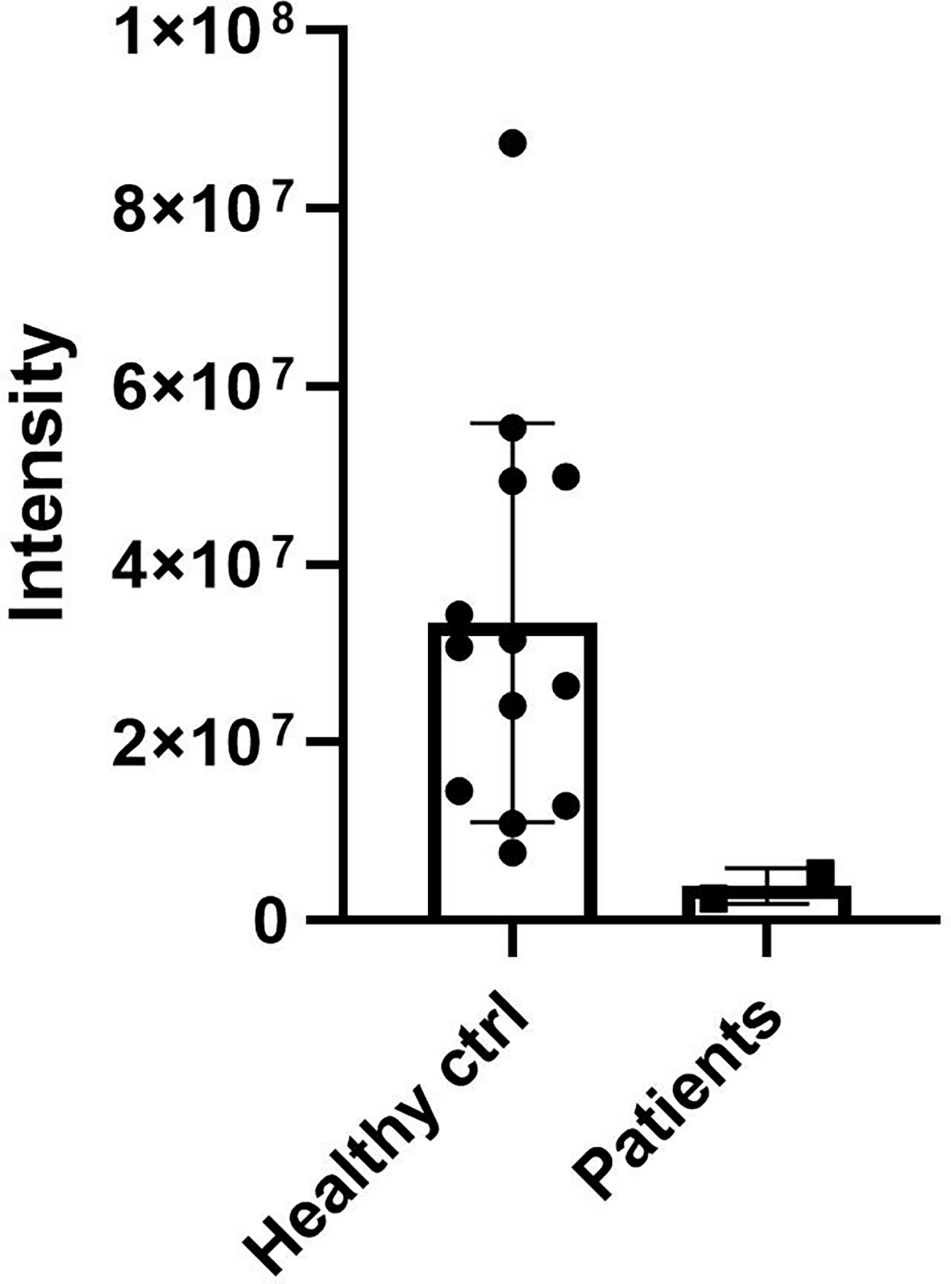
DCD levels in sweat of patients and controls. Sweat of 13 healthy controls (ctrl) and of Patient 1 and 2 (Patients) was analysed by nanoLC-MS/MS. The only common sequence (ENAGEDPGLAR) identified between patients and controls was used to determine the relative abundance of DCD. The graph shows the relative abundance of DCD peptide in sweat samples. Results represent the mean of three technical replicates for each sweat sample ± SD.

DCD_WT and DCD_MT peptides were initially digested in trypsin and analysed by nanoLC-MS/MS in order to identify proteotypic peptides. Subsequently, recovered sequences were searched in the sweat samples derived from 13 healthy controls and the two patients (Patient 1 and Patient 2) (**Supplementary Table 2**); the sweat retrieved from Patient 3 was not quantitatively sufficient for the analysis, so considering the age, 11 years, of the patient and the requirements of less invasiveness as possible from the Ethical Committee, we stopped the collection. Only one common sequence between patients and controls was identified (ENAGEDPGLAR), and it was therefore used to determine the relative abundance of DCD and to compare its level amongst all sweat samples. We observed a decrease of DCD levels in patients when compared to the control group (**Figure 2**). To estimate the level of MT_DCD peptide, we then used the peptide sequence DAVEDLESVGK, which is not included in the mutant, subtracting n the combined WT_DCD level. As a result, for Patient 2 the MT_DCD is present and it is roughly half of the total DCD protein (MT_DCD: 1.13 x106; MT+WT_DCD: 2.49 x106); notably it is clearly not present in the control group. Since DAVEDLESVGK peptide is much less abundant than the tryptic peptide ENAGEDPGLAR, we were able to measure this sequence only in Patient 2 sweat, since it was below the limit of detection of Patient 1 sample.

### 3.3 *In silico* analysis

#### 3.3.1 Comparative modelling

**Figure 3:**
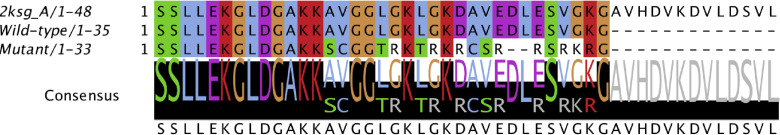
Alignment of the conserved domain of active dermcidin peptide. Conserved amino acid residues are highlighted. The black bars with letters at the bottom represent the conservation level and consensus sequence (with their possible amino acid variations). Structure of dermcidin human available on the Protein Data Bank (PDB ID: 2KSG).

For the wild-type and mutant sequences, the best-selected template was a structure of DCD from *Homo sapiens* (PDB code 2KSG), sharing 100% and 57.6% identity respectively. After stereochemical analysis of the amino acid sequences (**Figure 3**), it was possible to observe that 90% of the amino acid residues were in favourable positions for all models (**Supplementary Table 3**).

The Z-score indicated that all models were within the score range typically found for peptides of similar size. The qualitative model energy analysis distance constraints (QMEAN DisCo) score showed global energy of 0.67 for the wild type and 0.63 for the mutant.

#### 3.3.2 Molecular dynamics

In order to observe the modifications of the models after dynamics, the graphs of root mean square deviation (RMSD), root mean square fluctuation (RMSF), radius of gyration (Rg), and solvent accessible surface area, were evaluated (**Supplementary Figure 1**). All models left the original conformation (**Supplementary Figure 1A**), the wild type was stable for most of the molecular dynamics simulation (20 ns forward), while the theoretical structure model for the mutant took the time throughout the simulation to find stability similar to the wild type (50 ns forward). When the fluctuation of amino acid residues was analysed, the wild type showed greater flexibility, the b-factor revealed that the regions with greater dynamic mobility of the atoms were located either at the ends or in regions without secondary structure (loop) that generally have a higher degree of flexibility for fluctuation; in contrast, the mutant structure remained rigid when compared to the wild type peptide (**Supplementary Figures 1B, C**). The Rg (**Supplementary Figure 1D**) indicates that the wild type tends to acquire a compact structure, whereas the mutant moiety remains with few significant changes in the position of the mass axis. The solvent accessible surface area shows a greater accessible area for the mutant protein with a predilection for the C-terminus region if compared to the wild type AMP.

Although the models started from the same template, they present their own compaction and fluctuation characteristics, inferring the structural changes found in these analyses (**Supplementary Figure 2A**); the surface electrostatic potential was generated with the APBS tool (45) (**Supplementary Figure 2B**). The *in silico* results showed that the substitutions of amino acid residues in the mutant peptide significantly impact the structural characteristics of the AMP, specifically by rendering it more rigid and more favourable to interactions with the solvent. It is possible to speculate that the retrieved alterations in the structural behaviour of the mutant peptide might severely compromise its function when compared to the wild type AMP, either by modifying its mechanism of action or by inducing affinity towards novel targets.

#### 3.3.3 Principal component analysis

**Figure 4:**
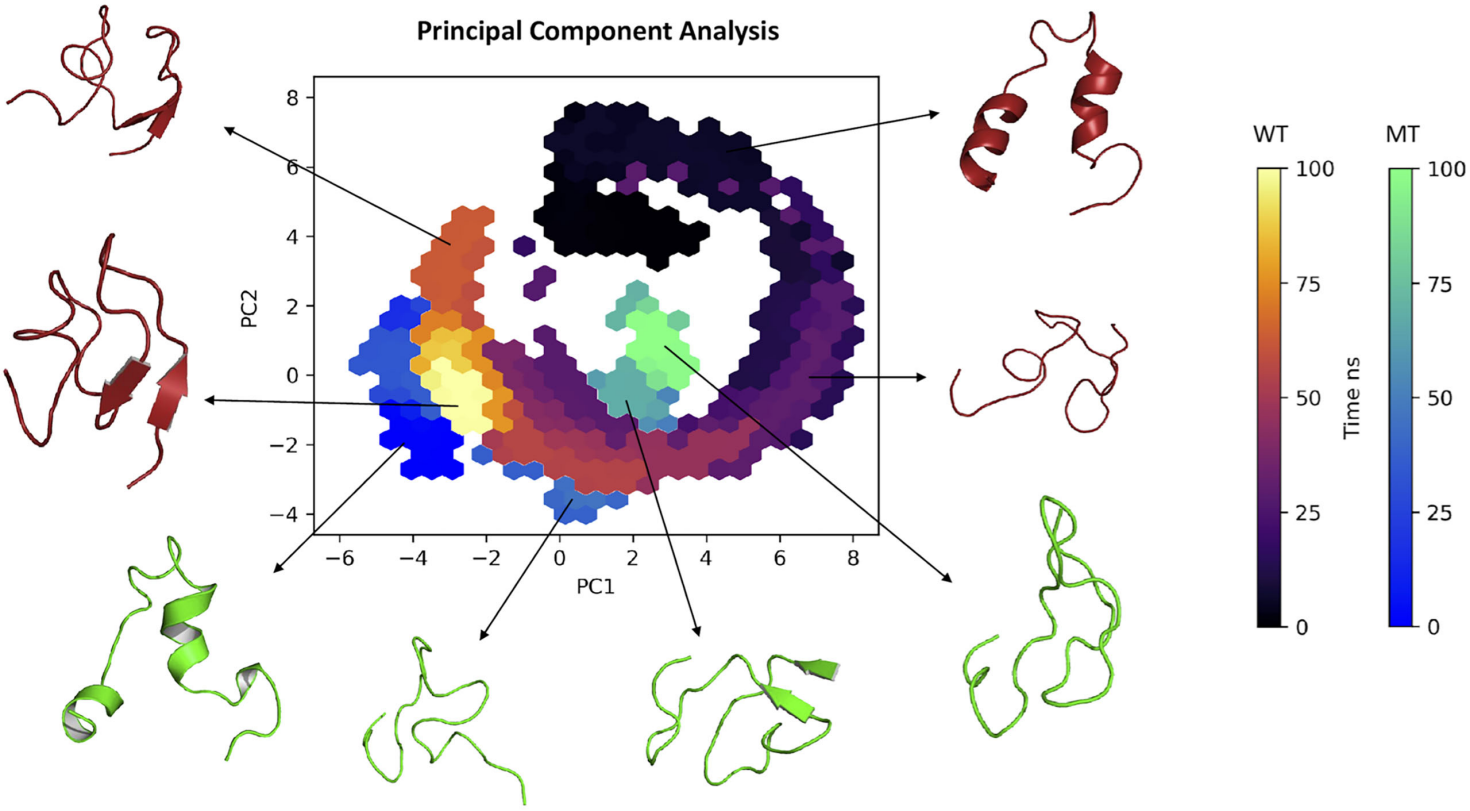
Principal component analysis (2D) clustering of various snapshots of the simulation trajectory based on respective Cartesian coordinates.

Regarding the PCA based on the trajectories of the simulation in molecular dynamics (a technique used to describe the conformational dynamics of the peptide through the atomic movements of the structure that are converted into main movements), **Figure 4** shows the clusters of conformations along the planes PC1 and PC2: we observed that the structure of the wild type in the 0 and 50 ns region showed a wide dispersion area along with the components, with considerable conformational variation and flexibility. On the other hand, movements between 75 and 100 ns, showed less dispersed values for both wild type and mutant converging to a more conserved cluster with less variation, but varying in length on the principal components. Interestingly, on PC1 wild type presented two different clusters in 50 and 75 ns before converging to a less dispersed stage.

### 3.4 Antimicrobial activity assays

**Figure 5:**
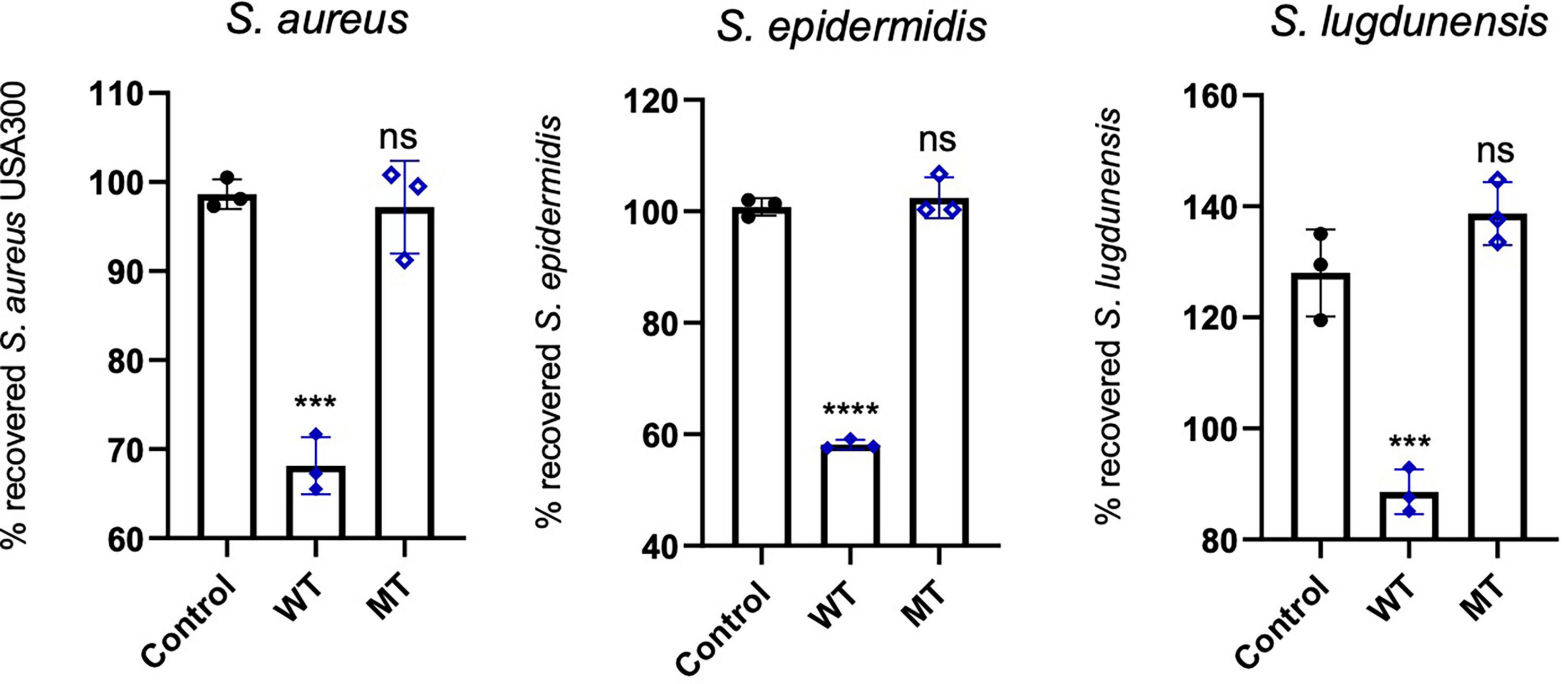
*In vitro* antimicrobial activity assay of DCD peptides. *S. aureus* USA300, *S. epidermidis* 1457 or *S. lugdunensis* (1x10^8^ CFU/mL) were incubated with 2 µM of human wild type (WT) or mutated (MT) DCD. After 3 hours of incubation, several dilutions of the bacterial suspensions were plated onto TSB agar plates and incubated overnight at 37 °C. The next day, bacterial colonies on an agar plate were counted and analysed using a colony-forming unit (CFU) assay. Results represent three independent experiments with four technical replicates each ± SD. Significant differences to control treatments were analysed by ordinary one-way analysis of variance (ANOVA) followed by Dunnett’s multiple comparisons test (ns: non significant; ***P<0.001; ****P< 0.0001).

We analysed the antimicrobial activity of wild type and mutated DCD peptides against the staphylococcal strains *S. aureus, S. epidermidis* and *S. lugdunensis* in a CFU assay. As shown in **Figure 5**, 2µM DCD_WT peptide exhibits a significant antimicrobial activity on all three bacterial strains, whereas the DCD_MT peptide had no significant effect on bacterial survival. These data indicate that the DCD mutation found in HS patients, resulting in the production of a truncated DCD peptide, elicit the loss of the antimicrobial activity of DCD.

**Figure 6:**
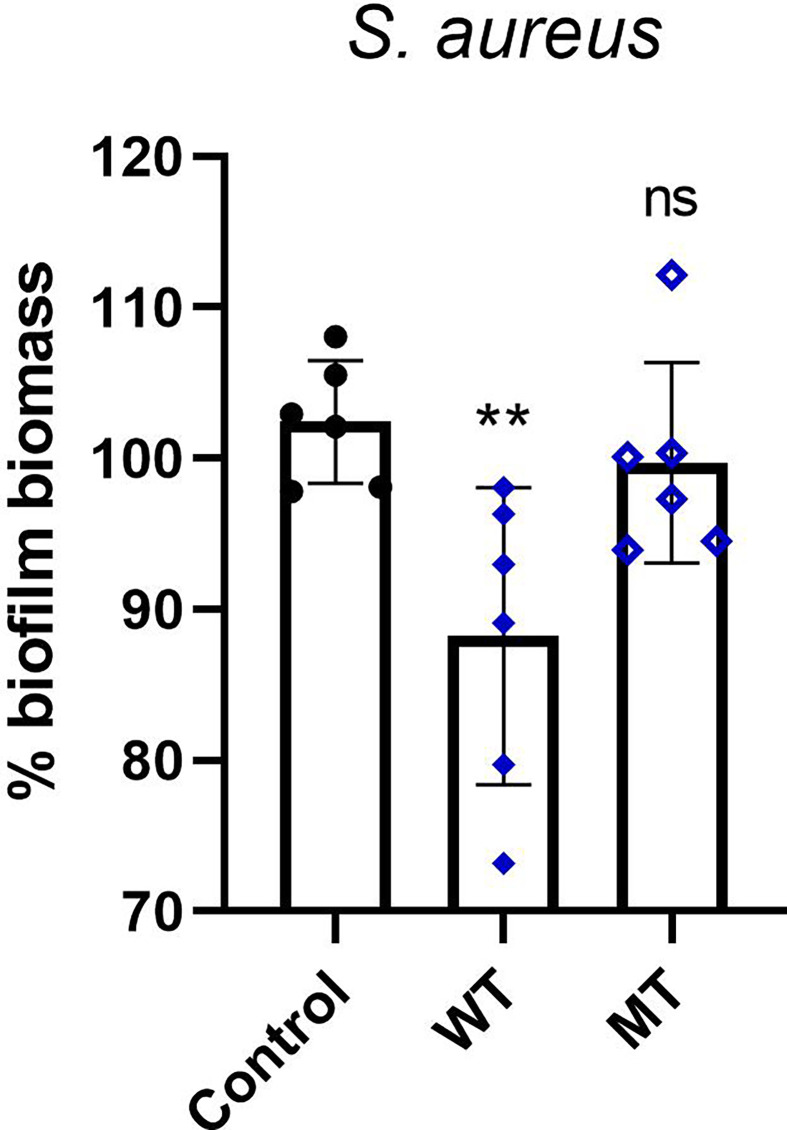
*In vitro* activity of DCD peptides AMPs against biofilm deposition. *S. aureus* (1 x 10^7^ CFU/mL) was incubated with 2 µM of human DCD_WT or DCD_MT peptide in BHI medium containing 2% sucrose and plated on 96-multiwell plates for 24 hours. Cells were fixed in methanol and stained with 0,1% crystal violet solution that was solubilised with 95% ethanol. The absorbance reads were measured at 560 nm using the GloMax^®^ Discover Microplate Reader (Promega). The low cut-off was represented by approximately 3 standard deviations above the absorbance mean of wells containing medium alone without bacteria. Results are represented as six independent experiments with 2 technical replicates each  ± SD. Significant differences to control treatments were analysed by ordinary one-way analysis of variance (ANOVA) followed by Dunn’s multiple comparisons test (ns: non significant; **P< 0.01).

Next, in *S. aureus* strain we evaluated the antimicrobial activity of DCD_WT and DCD_MT peptides against biofilm deposition; as indicated in **Figure 6**, DCD_WT peptide significantly inhibits biofilm deposition if compared to non-treated (Control) cells, but any significant changes in biofilm formation were registered in *S. aureus* samples incubated with DCD_MT peptide when compared to control cells.

### 3.5 Microbiome

**Figure 7:**
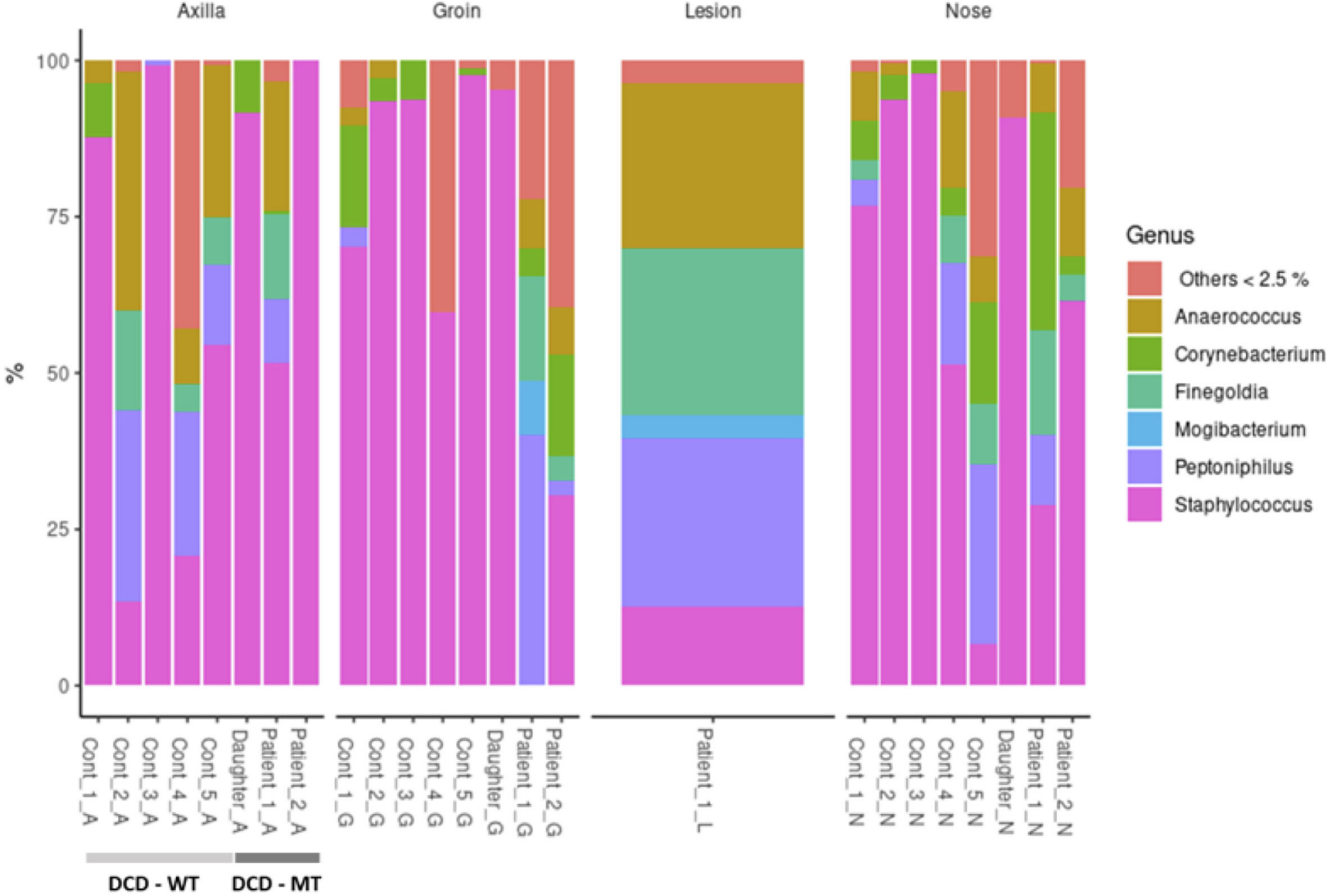
Taxonomic assignment of the most common genera (expressed as relative abundances) of study population (DCD-MT) samples versus healthy controls (DCD-WT) and stratified according to the anatomical site.

Usually, the composition of human microbiomes (alpha-diversity, reflecting richness and evenness of microbial taxa) varies significantly between individuals and body sites, and the inter-individual differences are frequently larger than the differences between sites of the same individual. Despite the small sample size encompassing one diseased individual (Patient 1), one individual with a history of the disease (Patient 2) and the 11-year-old daughter of Patient 1 - who presented isolated follicular lesions, then resolved spontaneously without flares -, we decided to investigate the microbiomes of the three individuals carrying the frameshift variant in *DCD*. As a control we used five healthy volunteers with no history of HS. Sampling sites were the anterior nares (N), the axillae (A), the groin (G), and for Patient 1 also a lesional site (L). Data analysis revealed no general differences between the samples of healthy controls and the three individuals carrying the heterozygous *DCD* mutation, neither on the phylum nor on the genus level (**Figure 7**).

All three sampling sites were dominated by Firmicutes and Actinobacteria with some samples containing Proteobacteria (**Supplementary Table 4**). On the genus level the diversity between the samples was clearer, showing that the daughter was dominantly colonised by various *Staphylococcus* species at all sites, but not by *S. aureus* (**Figure 7** and **Supplementary Table 4**). This pattern could also be found in healthy control 3 (Cont_3), although in this case the nasal sample was dominated by *S. aureus* (**Supplementary Table 4**). The most obvious differences resided in the microbiomes of the groin of Patient 1 and Patient 2 since there were either no *Staphylococcus* species detectable (Patient 1) or at comparatively low abundance (Patient 2). Instead, the groin of Patient 1 was heavily colonised by *Peptoniphilus*, also slightly detected in Patient 2, whereas only one control (Cont_1) expressed this genus in the groin. Additionally, Patient 1 had a high abundance of *Anaerococcus* and *Finegoldia magna* at all sites, which is the only species within this genus, and therefore differing from the controls. *Finegoldia magna* was detectable only in the groin samples of the two patients but not in those deriving from the controls. Noteworthy, *Peptoniphilus*, *Finegoldia* and *Anaerococcus* accounted for about 80% of species identified in the lesional site of Patient 1 and were also detected in the nose, the axillae and the groin (**Figure 7**). An obvious difference between the lesional site of Patient 1 and all other samples was also the presence of the rare genus *Mogibacterium*, which was expressed only at the lesional site and the groin of Patient 1. Again, this genus belongs to the strictly anaerobic growing Gram-positive cocci.

**Figure 8:**
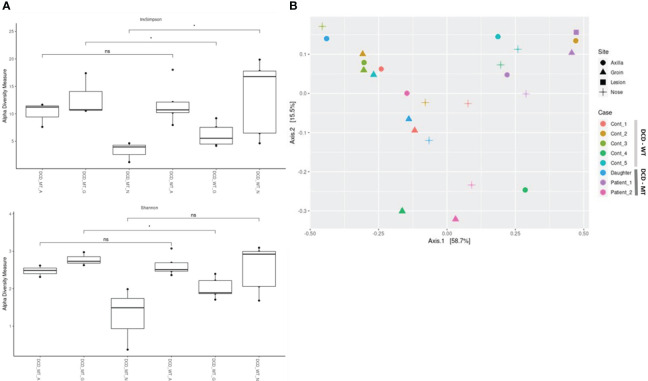
Characterization of microbial diversity in study population (DCD-MT) versus healthy controls (DCD-WT) in three anatomical sites **(A)** alpha diversity expressed in InvSimpson and Shannon measures and compared statistically using Wilcoxon signed-rank test **(B)** Beta diversity expressed in Weighted UniFrac measure and Principal Coordinate Analysis (PCoA). (ns: non significant; *P< 0.05).

Alpha-diversity of the DCD-negative individuals and the healthy controls was analysed *via* Inverse Simpson, Shannon (**Figure 8**) and Chao1 (**Supplementary Figure 3**) and compared using Wilcoxon signed-rank test. These analyses indicated a small but significant difference between the groins of DCD_WT and DCD_MT samples with a higher diversity in the DCD-negative samples (**Figure 8A**). Similarly, but significant only in the inverse Shannon analysis, the nasal microbiomes of the two groups exhibited different alpha-diversity. Here, DCD-negative samples showed a lower diversity. There was no difference in the alpha-diversity of axilla samples. This might indicate that the loss of dermcidin production has a site-specific impact on the microbiome diversity (**Figure 8A**), nevertheless for a definite conclusion the sample size of three individuals might be too small.

The principal coordinate analysis (PCoA) of all samples reflecting beta-diversity did not show clustering of the DCD-negative individuals (**Figure 8B**). Only the four samples of Patient 1 showed some clustering due to the presence of *Peptoniphilus*, *Finegoldia* and *Anaerococcus* at all tested body sites.

Finally, differential abundance analysis revealed the species which significantly differ between the corresponding sites of DCD-WT and DCD-MT groups. As already highlighted, *Finegoldia magna* and *Peptoniphilus* sp. are clearly associated with the groins of the DCD-MT cluster, whereas the presence of *Staphylococcus caprae* is linked to the groins of DCD-WT group (**Table 1**). The second highly significant difference affects the nasal samples where *Dolosigranulum pigrum* is associated with DCD-WT and *Bosea vestrisii* with DCD-MT.

**Table 1:** Identified differential abundant taxa (adjusted P value <0.05) when comparing groin and nasal microbiomes of DCD_MT study population versus healthy controls (DCD_WT) using Deseq2.

Species	Phylum	Padj value	log2FoldChange	Associated with
*Finegoldia magna*	Firmicutes	0.000419195	7.823801893	DCD_MT_(Groin)
*Peptoniphilus lacrimalis*	Firmicutes	0.000474896	7.00737648	DCD_MT_(Groin)
*Peptoniphilus* sp.*_HMT_375*	Firmicutes	0.000474896	6.978378646	DCD_MT_(Groin)
*Staphylococcus caprae*	Firmicutes	0.001958727	-8.157705432	DCD_WT_(Groin)
*Dolosigranulum pigrum*	Firmicutes	0.001116527	-8.044364768	DCD_WT_(Nasal)
*Bosea vestrisii*	Proteobacteria	0.010531636	5.209435852	DCD_MT_(Nasal)

## 4 Discussion

HS is an autoinflammatory skin disease with a multifactorial aetiology that involves a strict interplay between genetic factors, immune dysregulation and lifestyle factors. Familial forms represent 40% of the total HS cases and show an autosomal dominant, but with incomplete penetrance, mode of inheritance of the disease (47). By applying WES analysis in a family affected by HS we found a rare frameshift insertion in exon 4 of *DCD* gene in heterozygosis (rs538180888; g.54645236_54645237insT; NM_053283.4:c.225dup; NP_444513.1:p.Ala76Fs) in both HS cases (Patient 1 and Patient 2) and in the 11-year-old daughter of Patient 1, while the identified variant was not detected in the mother of Patient’s 1 daughter, healthy control who reported not to be affected by any known disease. Our results highlight that the frameshift insertion is carried in heterozygosis by Patient 1, Patient 2 and in Patient’s 1 daughter, thus suggesting an autosomal dominant inheritance pattern that is generally observed in HS familial cases (3).

It should be considered that the daughter is a pre-adolescent and, to date, she presented just an isolated occurrence of follicular lesions, which resolved spontaneously without flares. Noteworthy, data concerning the adolescent-onset of HS are scarce and the percentage of cases bearing an adolescent outbreak of the disease appear to change from 2% under 11 years of age to 38.3% under 17 years of age (48–50). Therefore, it is possible to speculate that the daughter might be diagnosed with HS in the next few years.

*DCD* gene encodes for dermcidin (DCD), an antimicrobial peptide (AMP) possessing no homology to other known AMPs and firstly described by Schittek B. et al. (19). DCD was originally defined as an AMP that is specifically and constitutively expressed in the sweat gland, and that undergoes proteolytic processing when secreted into eccrine sweat. Once activated by proteolytic cleavage, the active moiety of DCD is transported through sweat to the epidermal surface, site in which the compound actively participates in creating a first line of cutaneous innate immune defence by generating a protective barrier spread over the epidermis (19) that exerts a potent antimicrobial activity by forming ion channels in the membrane of pathogens, hence resulting in bacterial cell death (51, 52).

Considering that the expression of DCD is specifically limited to sweat, DCD levels were measured in sweat samples deriving from Patient 1, Patient 2 and in healthy controls. We observed a reduction of DCD abundance, particularly of a common fragment found both in the wild type and mutated DCD peptide, in patients when compared to the control group. Recent skin transcriptome analyses, conducted by Coates M. et al. (11), reported a downregulation of DCD in HS lesions. In detail, they analysed publicly available microarray dataset of lesional and non-lesional skin of HS patients, also compared to RNA-seq dataset of wounded and non-wounded human skin samples. These data revealed an upregulation of DCD in wounded skin and a downregulation in HS skin, data that were all also confirmed by RT-qPCR and immunofluorescence in skin biopsies (11).

Another skin transcriptome analysis, conducted by Shanmugam V.K. et al. (16), confirmed a downregulation of DCD in HS skin. In detail, they observed a significant downregulation of DCD in skin from 10 HS patients compared to 11 normal skin harvested at abdominoplasty. Our results, obtained by measuring the DCD levels directly in sweat, confirmed these previous observations, despite the low number of analysed samples.

The observed down-secretion of DCD suggests that insufficient levels of this AMP could lead to bacteria overgrowth and microbial dysbiosis found in HS (53). AMPs in general are important in the control and management of the microbiome’s composition, therefore alterations in AMPs levels could cause dysbiosis (54). All these findings suggest that regulators of the innate immune response, in particular DCD, may play a pivotal role in the pathogenesis of HS.

Our *in silico* studies show that the identified frameshift variant disrupts the ORF of *DCD* and results in a truncated 33 amino acid peptide having a completely altered sequence from position 15 to 33 if compared to the wild type DCD peptide. As a consequence, the alteration of the sequence affects both the N-terminal and the C-terminal partitions of the AMP. Indeed, in the mutated sequence it is possible to observe an overall increment in positively charged amino acids and a reduction in the negatively charged ones. It is possible to speculate that the resulting imbalance in the net charge of the molecule might lead to the loss of the amphiphilic nature of the peptide, hence causing severe repercussions on the peptides’ antimicrobial activity (52). Specifically, mutated DCD might either affect the electrostatic interactions occurring between the cationic N-terminal domain and the negatively charged bacterial phospholipids, the formation of oligomeric complexes on the bacterial surface or the formation of the membrane-embedded ion channel.

Observing the dynamics of the 3D structure of the DCD peptide, unlike the wild type AMP,mutant DCD presents a rigid structure, with a tendency towards a major stability. The mutant peptide shows a minor structure compaction and an increased solvent accessibility which exhibits greater atomic movements, especially in the C-terminal region, while the wild type molecule shows greater movement in the N-terminal region (**Supplementary Figure S1**). A principal component analysis of the pathways of the peptides highlighted a separation of the two structures (wild type and mutant) into different components, with larger dynamic shifts to the wild type, confirming the impact on the structure stability of the mutant form during the simulation. These structural differences caused by mutations in amino acid residues may cause a decrease in the activity of the mutated peptide, since the structure determines the function and this relationship is governed by the natural selection process (55, 56).

In order to confirm the *in silico* results we performed *in vitro* antimicrobial assays. We observed that DCD_MT peptide did not induce a significant change in the planktonic growth of *S. aureus*, *S. epidermidis*, *S. lugdunensis* as opposed to DCD_WT which significantly reduced the planktonic growth of all tested bacterial strains if compared to non-treated bacteria (**Figure 5**). We also found that DCD_MT peptide, as opposed to DCD_WT AMP, is unable to induce alterations in biofilm deposition *S. aureus* samples (**Figure 6**). Collectively, these results demonstrate that the structural differences between DCD_WT and DCD_MT peptides cause an important decrement in the antimicrobial activity of this AMP.

Considering that a reduction in the quantity and in the antimicrobial activity of DCD could cause alterations in the microbial populations, we decided to perform microbiome analysis in recruited patients. The microbiome of HS cases has been previously investigated. Whereas no significant differences were found in the bacterial richness (alpha-diversity) between non-lesional sites of HS patients and healthy controls, 14 taxa were statistically different between HS and healthy samples. Among these, *Prevotella*, *Actinomyces*, *Campyobacter ureolyticus*, and *Mobiluncus* showed higher abundance in HS samples, whereas skin commensals such as *Staphylococcus epidermidis*, *Micrococcus luteus* and *Kocuria* exhibited lower abundance (57). In contrast, lesional skin sites of HS patients were dominated by *Corynebacterium* spp., and anaerobic *Porphyromonas*, and *Peptoniphilus* spp (58). In our study the currently diseased Patient 1 showed a very similar pattern within his microbiome. Whereas the abundance of *Staphylococcus ssp.* was generally low or even absent in the groin, *Peptoniphilus*, but also its close relatives *Finegoldia magna* and *Anaerococcus*, dominated the microbiomes, especially in the lesional site. All three genera are anaerobically growing bacteria and belong to the family of Peptoniphilaceae. In fact, all three genera were formerly classified in the genus *Peptostreptococcus*. In this study we identified *Mogibacterium* at the lesional site of Patient 1, which is also closely related to the aforementioned anaerobe Gram-positive cocci.

Furthermore, to investigate a putative impact of dermcidin on the various microbiomes we compared DCD_MT and DCD_WT samples. Analysis of alpha-diversity revealed a significant difference between the groins of DCD_MT and DCD_WT groups. Therefore, we speculate that dermcidin might play a role only at specific body sites, depending on the level of expression or on site-specific physico-chemical conditions. At the groin, which usually shows high humidity and salinity, dermcidin production might be responsible for the lower diversity in DCD-WT individuals.

## 5 Conclusions

The rare frameshift insertion in *DCD* gene found in a small HS family caused structural differences in dermcidin that induced a loss of the antimicrobial activity of this AMP which could lead to an alteration in microbial populations found in HS. In these HS patients, we also confirmed a downregulation of DCD peptide levels as already observed in other studies. We are aware that our study suffers technical limitations due to the different and much shorter sequence of the mutant dermcidin peptide, so we have not found a reliable method to directly measure only mutant DCD peptide in the sweat.

All of this evidence led us to hypothesise a potential role of DCD in HS etio-pathogenesis and suggested that insufficient levels of this AMP may induce microbial skin alterations. Being aware that AMPs other than DCD play a role in HS etio-pathogenesis, restoring physiological levels of DCD in HS patients could be very interesting ansinced this molecule could constitute a novel target for personalised therapeutic approach. How to restore normal DCD level is still an open question; we can figure out a topical and personalised treatment exploited by a progressive delivery of DCD in cases lacking this antimicrobial peptide.

## Data availability statement

The raw datasets analyzed for this study can be found in the NCBI’s SRA database (id:SAMN30804682; SAMN30804683; SAMN30804684). The detailed result of the microbiome analyses is in **Supplementary Table 2**. Additional information can be obtained from the corresponding authors upon reasonable request.

## Ethics statement

The studies involving human participants were reviewed and approved by Comitato Etico Unico Regionale (CEUR) of Friuli Venezia Giulia (FVG). Written informed consent to participate in this study was provided by the participants’ legal guardian/next of kin.

## Author contributions

PT and SC conceived and planned the experiments. PT, RG and BU performed the samples preparation. CS-S performed the *in silico* experiments. RM performed the WES analyses. ES and PC performed the sweat analysis. AE and BK carried out the microbiome analyses. SC and CM performed genetic counselling. IB and KC provided the clinical data. RG, CDV, LS and BS carried out the antimicrobial assay. PT, RG, AD'A, MB and SC contributed to the interpretation of the results. PT, RG, CS-S, BK, BS and SC wrote the original draft manuscript. All authors contributed to the article and approved the submitted version.

## Funding

This work was supported by a Biomolecular Analyses for Tailored Medicine in AcneiNversa (BATMAN) project, funded by ERA PerMed (JTC_2018), by Starting Grant (SG-2019- 12369421) founded by the Italian Ministry of Health, by grants (RC16/2018 and RC03/2020) from the Institute for Maternal and Child Health IRCCS ‘Burlo Garofolo funded by the Italian Ministry of Health. Furthermore, this work was supported (BS and BK) by the Cluster of Excellence EXC2124 project ID -390838134 of the University of Tübingen and the German Center of Infection Research (DZIF) (BK).

## Acknowledgments

The authors acknowledge support by the High Performance and Cloud Computing Group at the Zentrum für Datenverarbeitung of the University of Tübingen, the state of Baden-Württemberg through bwHPC and the German Research Foundation (DFG) through grant no INST 37/935-1 FUGG. **Figure 1** was created with BioRender.com.

## Conflict of interest

The authors declare that the research was conducted in the absence of any commercial or financial relationships that could be construed as a potential conflict of interest.

## Publisher’s note

All claims expressed in this article are solely those of the authors and do not necessarily represent those of their affiliated organizations, or those of the publisher, the editors and the reviewers. Any product that may be evaluated in this article, or claim that may be made by its manufacturer, is not guaranteed or endorsed by the publisher.
