# Supplementary material for: A rare loss-of-function genetic mutation suggest a role of dermcidin deficiency in hidradenitis suppurativa pathogenesis
Source: Front Immunol. 2022 Dec 5;13:1060547. doi: 10.3389/fimmu.2022.1060547 (PMC9760663; doi:10.3389/fimmu.2022.1060547)
Supplement: Supplementary file 1 [file DataSheet_1.docx]

Supplementary Material

#
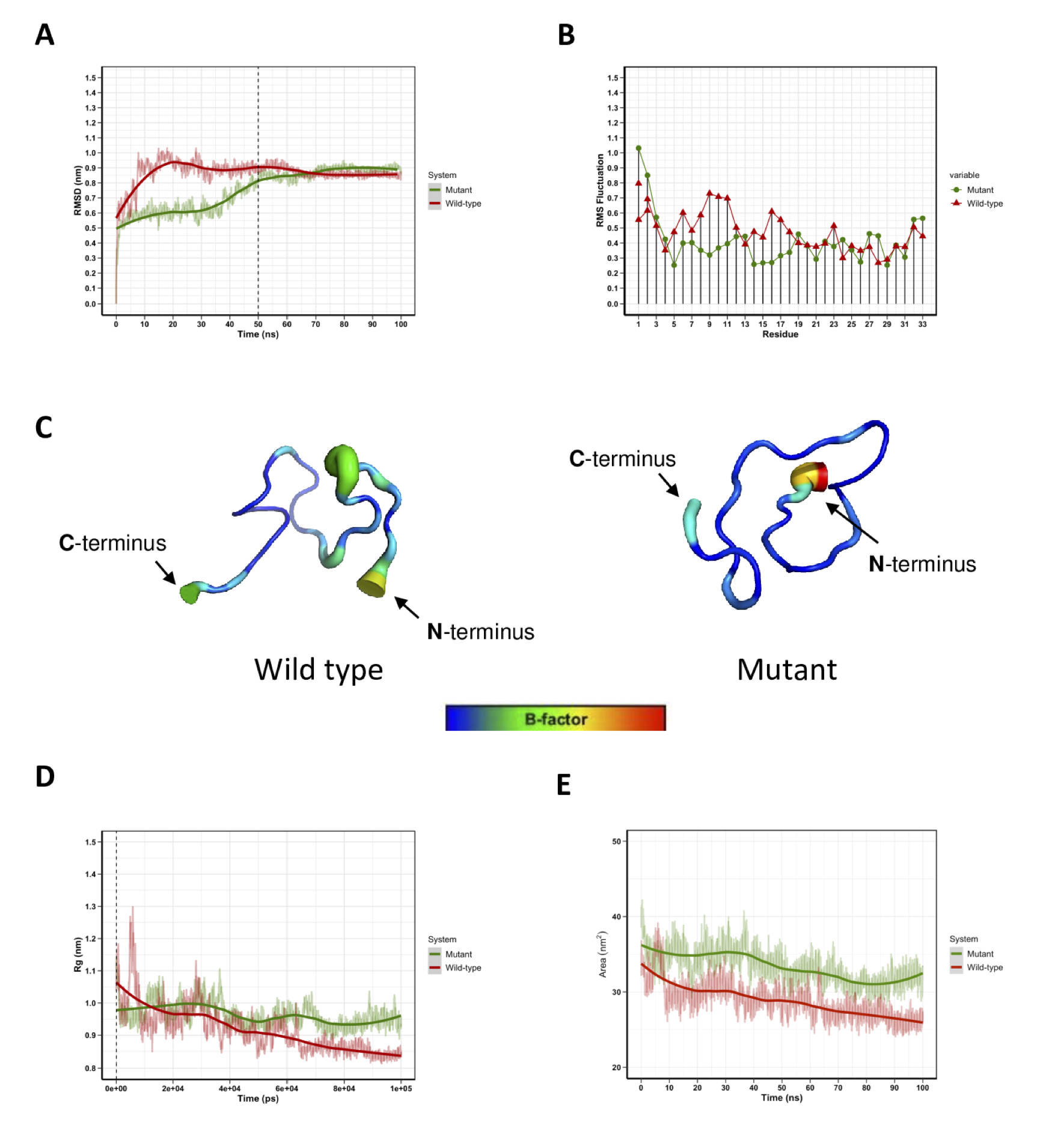
Supplementary Figures

# Supplementary Figure S1. Molecular dynamics simulations of the theoretical three-dimensional models wild type (in red) and mutant (in green). (A) RMSD showing the deviation of the theoretical models after the dynamics. In B the RMSF of the models represented by the fluctuation of amino acid residues concerning the time of 100 ns. (C)  Representation of DCD corresponding to the b-factor or scattering of atoms of the three related models, the closer to the red, the greater the dynamic mobility of the atoms, and the closer to the blue, the less dynamic mobility of the atoms. (D) Radius of gyration showing the compaction of the models and E. Solvent accessible surface area.


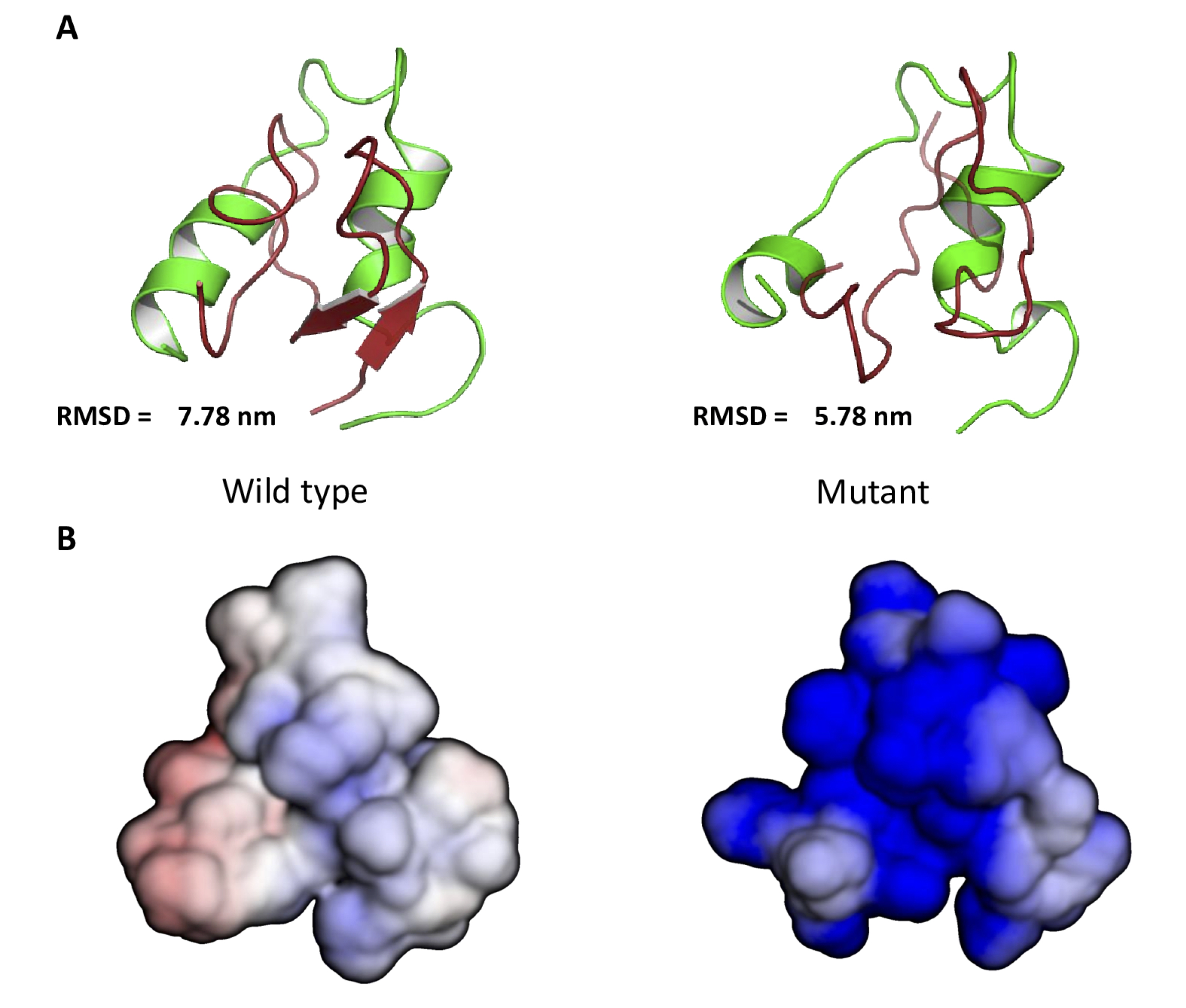


#
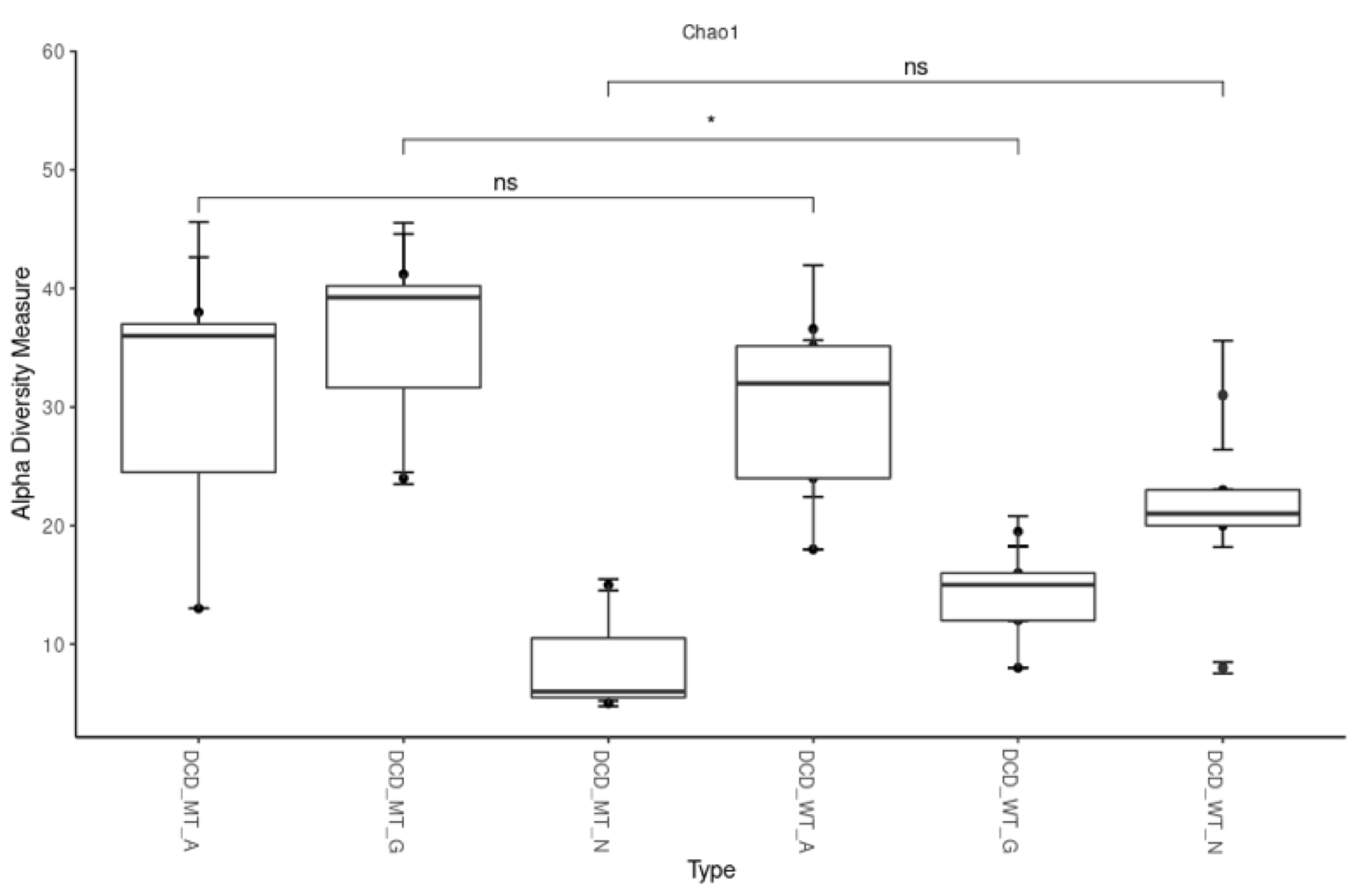
Supplementary Figure S2. Three-dimensional models of DCD. (A) Above the 3D structures the alignment of the primary sequences between wild type and mutant, highlighting the conserved amino acid residues between them. Below the alignment, 3D structures before (green) and after (red) the simulation in molecular dynamics with the respective distance (in nanometres) between the models. (B) The electrostatic surface potential (generated with APBS) plotted on the surface accessible by solvent for the theoretical three-dimensional models of DCD, showing the predominance of cationic charges surface for mutant and an amphipathic surface for wild type.

# Supplementary Figure S3. Characterization of microbial diversity in study population (DCD-MT) versus healthy controls (DCD-WT) in three anatomical sites alpha diversity expressed in Chao1 measures and compared statistically using Wilcoxon signed-rank test. (*P < 0.05).

## Supplementary Tables

# Supplementary Table 1. All genetic variants present in Patient 1 and Patient 2 and not present in mother.

# Supplementary Table 2. DCD derived peptide sequences detected.

| **Peptide Sequence** | **Protein Accessions** | **Charge** | **MH+ [Da]** | **RT [min]** |
| --- | --- | --- | --- | --- |
| AVGGLGKLGK | P81605 | 2 | 899.5664 | 13.3907 |
| AVGGLGKLGKDAVEDLESVGK | P81605 | 3 | 2042.111 | 21.7946 |
| DAVEDLESVGK | P81605 | 2 | 1161.563 | 17.8477 |
| DAVEDLESVGKGAVHDVK | P81605 | 3 | 1867.941 | 19.1286 |
| ENAGEDPGLAR | P81605 | 2 | 1128.528 | 11.6149 |
| GAVHDVKDVLDSVL | P81605 | 2 | 1466.787 | 20.827 |
| KAVGGLGKLGK | P81605 | 2 | 1027.662 | 11.8659 |
| LGKDAVEDLESVGK | P81605 | 2 | 1459.764 | 17.3373 |
| LGKDAVEDLESVGKGAVHDVK | P81605 | 4 | 2166.144 | 16.5345 |
| SSLLEKGLDGAK | P81605 | 2 | 1217.674 | 15.3424 |
| SSLLEKGLDGAKK | P81605 | 3 | 1345.77 | 13.9347 |

### Supplementary Table 3. Results of the algorithms for modelling and validation of the theoretical models of dermcidin.

| **ID** | **Template** | **Identity** | **Qmean_Global Score** | **Ramachandran*** | **Z-Score** |
| --- | --- | --- | --- | --- | --- |
| Wild-type | 2ksg.A | 100% | 0.84 ± 0.12 | 96% | -5.63 |
| Mutant | 2ksg.A | 57.6% | 0.79 ± 0.12 | 93% | -4.99 |

* Most favored regions

### Supplementary Table 4.  All genera/species of bacteria identified in all samples.
